# Statistical relationships between journal use and research output at academic institutions in South Korea

**DOI:** 10.1007/s11192-015-1563-0

**Published:** 2015-03-26

**Authors:** Youngim Jung, Jayhoon Kim, Minho So, Hwanmin Kim

**Affiliations:** 1 KISTI, 245 Daehangno, Yuseong-gu, Daejeon, 305-806 Republic of Korea; 2 KAIST, 291 Daehangno, Yuseong-gu, Daejeon, 305-701 Republic of Korea

**Keywords:** Correlation analysis, Comparative analysis, Diachronic analysis, e-Journal use, Research output

## Abstract

In this study, we analysed the statistical association between e-journal use and research output at the institution level in South Korea by performing comparative and diachronic analyses, as well as the analysis by field. The datasets were compiled from four different sources: national reports on research output indicators in science fields, two statistics databases on higher education institutions open to the public, and e-journal usage statistics generated by 47 major publishers. Due to the different data sources utilized, a considerable number of missing values appeared in our datasets and various mapping issues required corrections prior to the analysis. Two techniques for handling missing data were applied and the impact of each technique was discussed. In order to compile the institutional data by field, journals were first mapped, and then the statistics were summarized according to subject field. We observed that e-journal use exhibited stronger correlations with the number of publications and the times cited, in contrast to the number of undergraduates, graduates, faculty members and the amount of research funds, and this was the case regardless of the NA handling method or author type. The difference between the maximum correlation for the amount of external research funding with two average indicators and that of the correlation for e-journal use were not significant. Statistically, the accountability of e-journal use for the average times cited per article and the average JIF was quite similar with external research funds. It was found that the number of e-journal articles used had a strong positive correlation (Pearson’s correlation coefficients of *r* > 0.9, *p* < 0.05) with the number of articles published in SCI(E) journals and the times cited regardless of the author type, NA handling method or time period. We also observed that the top-five institutions in South Korea, with respect to the number of publications in SCI(E) journals, were generally across a balanced range of academic activities, while producing significant research output and using published material. Finally, we confirmed that the association of e-journal use with the two quantitative research indicators is strongly positive, even for the analyses by field, with the exception of the Arts and Humanities.

## Introduction

Usage statistics data by themselves are not a new phenomenon (Matthews 2009). Traditionally, libraries have maintained statistics records for gate counts, circulation, inter-library loans, and information services (NISO 2007). Academic libraries also frequently gather statistics on reserve items and submit reports to government agencies, parent institutions and library boards. Such information can then be used to understand work flow and improve library services. Since the rapid emergence of electronic resources and the explosion of online services becoming available through the internet, many institutions have acknowledged the benefits of investing in the collecting, reporting and analysis of usage statistics (Welch 2005). Usage statistics for online resources have also been a primary analytical data source for public and private library consortia. The Virtual Library of Virginia consortia have undertaken a project to streamline, automate and standardize statistical data collection and the reporting process (Matthews 2009). Joint Information Systems Committee has been developing the Journal Usage Statistics Portal providing a ‘one-stop-service’ for librarians to view, download and analyze their usage reports. Since 2009, the Korea Institute of Science and Technology Information (KISTI) has consolidated the usage statistics of member institutions in order to provide integrated usage data with subscription and bibliographic information to members, as well as to monitor the use of e-journal packages distributed through the Korea Electronic Site Licensing Initiative (KESLI) consortia (Jung et al. 2013).

Recently, a number of researchers have attempted to measure scientific or research impact from usage data available online from publishers, aggregators, electronic resource management systems and link resolvers (Bollen et al. 2009). Larsen and Von Ins (2010) argued that the coverage of SCI has declined with the rapid growth of scientific publications. Existing citation databases such as WoS, Scopus and Google Scholar have limitations in coverage and depth for non-English language journals. Zainab et al. (2012) and Choi et al. (2013) argue that WoS and Scopus are comprehensive databases for English journals but do not adequately cover most national journals published in developing countries.

Usage data regarding PDF downloads, HTML views and SNS saves are considered as valuable, and complement traditional citation-based assessment metrics. The PLoS Medicine Editors (2006) predicted that new measures including the number of times downloaded, coverage in mainstream media, and times referenced in policy documents might be useful inputs to measure the impact of rarely cited but influential articles. “Altmetrics” builds on information from social media use, and is suggested to present a nuanced and multidimensional view of multiple research impact measures over time, together with traditional citation-based metrics (Priem and Piwowar 2012). Bollen et al. (2009) analyzed and compared citation and usage networks, as well as various social network and hybrid measures to explore the most suitable methods for expressing and interpreting scientific impact. As a result, the ‘Usage Closeness Centrality’ incorporating 39 measures was deemed the best candidate for determining a ‘consensus’ view of scientific impact. Priem and Piwowar (2012) demonstrated that PDF downloads and HTML views correlate at moderate to high levels with almost all other Altmetrics. In particular, a moderately strong relationship between citation count and PDF/HTML download count for the three journals analyzed was reported in the study.

Brody and Harnad (2005) postulated that if a correlation exists between citations and downloads, a higher rate of downloads in the first year of an article could be used to predict a higher number of eventual citations later. They identified an overall correlation of 0.4 between the citation numbers and download impact of articles in physics and mathematics archived in the UK’s arXiv.org. Moed (2005) determined that during the first 3 months after an article is cited, the number of downloads increased 25 % compared to what would be expected had the article not been cited. In contrast, no relation between the usage factor and two measures of citation impact including the Journal Impact Factor and Elsevier’s Source Normalized Impact per Paper (SNIP) metric was reported (CIBER Research Limited 2011). Nevertheless, this particular report concluded that the new usage-based metric opens up the possibility of developing new ways of looking at scholarly communication, with different journals occupying very different niches within a complex ecosystem. Two reasons for the importance of download impact were highlighted by Brody and Harnad (2005): (1) The proportion of download variance correlated with citation counts provides an early estimate of probable citation impact that can be tracked from the instant an article is made Open Access, and attains its maximum predictive power after 6 months; and (2) The portion of download variance that does not correlate with citation counts provides a second, partly independent estimate of the impact of an article, sensitive to another form of research usage that is not reflected in citation counts. In the same context, efforts to develop and substantiate usage-based metrics have been undertaken by several research groups (Bollen and Van de Sompel 2008; Gorraiz and Gumpenberger 2010) as well as the Counting Online Usage of NeTworked Electronic Resources (COUNTER) group (Shepherd 2011).

It has generally been assumed that economic factors such as Gross Domestic Product (GDP), Gross Domestic Expenditures on Research and Development (GERD), and human resources have a linear or exponential relationship with research performance at the national level (Yoon 2007; Vinkler 2010). According to Wood (1990), personal characteristics and differences in research style, processes and techniques for research, as well as funding availability are the most important factors influencing the research productivity of academics. Heavy teaching loads were seen as a distraction from research activity in general. In particular, for the majority of science departments in academic institutions, the extent and continuation of funding are critical for facilitating research and many serious problems arise due to funding restrictions. However, to the best of our knowledge, there have been no previous studies investigating the statistical relationship between the production of resesarch publications and the use of them at the national or institutional level. Although the use of research publications is a daily necessity for researchers, investigations into the relationship between the use and production of research output at the institutional level in comparison to studies based on journal-specific metrics have been lacking.

In this study, our objective is to explore the relationship between research performance and the usage of publications at the level of institutions by performing comparative, diachronic analyses and an analysis by field. Specifically, we address the following three research questions:

1. Which is most highly correlated with research output at the institutional level: human resources, economic factors or the use of research publications?
2. Does the extent of correlation of e-journal use with four research output measures for long term data differ when assessed from a short term perspective?
3. What is the statistical relationship between the use of research publications and research productivity at the institutional level (by field)?

## Data, methodology and limitations

### Dataset

In order to address our research questions, we referred to SCI Analysis Research, an annual report on national science and technology indicators covering a range of different scientific fields, research institutions, journals and regions in South Korea, published by the National Science and Technology Commission (NSTC). The reports are compiled as a result of search and analysis of the Web of Science (WoS) and NSI databases every year. They include the number of articles published in Science Citation Index [SCI(E)] journals, times cited, average times each article is cited and average journal impact factor (JIF)^1^ as research performance indicators for each institution (NSTC 2011). The institutional statistics are calculated according to author type in three ways: (1) by quantifying every affiliation present in the author list in each article (Vinkler 2010), (2) by accounting for the affiliation of the first author only, and (3) accounting for the affiliation of the corresponding author only.

Neither the NSTC report nor NSI databases include institutional statistics related to human resources, research funding and journal use. We therefore decided to collect the relative institutional data from several different data sources. In order to obtain institutional statistics on human resources and research funds, we used two websites available to the public; Statistics of Korean Universities managed by the Korea University Accreditation Institute (KUAI),^2^ which provides the statistics for the number of undergraduates and graduate students, and the average internal/external research funding available per faculty member. The number of faculty members at Korean academic institutes is recorded by the Korea Educational Statistics Service (KESS^3^), operated by the Korea Education Development Institute (KEDI).

The usage statistics for e-journals by academic institutions are generated and managed by each content provider and are not publicly available. The KISTI manages the KESLI consortia, the biggest library consortia in South Korea, and has developed an automated collection system for e-journal usage statistics from 47 major content providers on behalf of member libraries (Jung and Kim 2013). The publisher-generated statistics for 522 institutions are collected automatically on a monthly basis, and include Elsevier, Springer, Wiley, Nature Publishing Group, the American Association for Advancement of Science (AAAS), the American Chemical Society, the Institute of Electrical and Electronics Engineers, American Institute of Physics, American Physical Society, Institute of Physics and others. The data format follows Release 3 of the COUNTER code of practice for e-journals, Journal Report 1 (JR1), where ‘total use’ denotes the sum of HTML views and PDF downloads (COUNTER 2008).^4^

Most major content providers provide COUNTER-compliant usage statistics data for their clients, however it is not very common for a publisher to provide such usage data stretching back considerably over many years. The more prolific publishers and those with a higher readership necessarily incur greater costs to maintain extensive records of past usage data. The International Coalition of Library Consortia (ICOLC) recommends that publishers maintain a minimum of 3 years of such historical data. These data should ideally be made available in separate files containing specified data elements that can be downloaded and manipulated locally (ICOLC 2006). Most major publishers follow this recommendation and provide several years of past usage data. Thanks to KISTI’s automated collection of e-journal usage data on behalf of the majority of academic institutions in South Korea since 2009, the usage data for several publishers including AAAS, the National Academy of Science and Berkeley Electronic Press have been recorded since 2000, and the past usage data for most content providers is available for 2007 onwards (Jung and Kim 2013).

The data obtained from the four different sources were merged based on the NSTC reports. Only the institutional data appearing in the NSTC reports were considered for the analysis. According to the NSTC report, the number of articles authored by Korean researchers totaled 41,114 in 2010, and 270,420 for the 10 years from 2001 to 2010. The number of institutions that published at least one article in an SCI(E) journal decreased in relation to author type from 292 to 202. The three datasets generated according to author type were used to address the first and the second research questions. Only the number of publications in SCI(E) journals and the times cited are available as research output indicators in the corresponding author dataset. Table 1 presents the explorative description of data used for the 2010 analysis according to author type.

**Table 1:** 2010 Sample description (e-journal use in 2008, 2009 and 2010)

Variable	*N*	Min	1st Q.	Med	Mean	3rd Q.	Max	NAs
Co-author basis								
# Publications	292	1	2	8	181.5	96.8	5516	–
Times cited	292	0	0	4	195.6	55.8	7424	–
Avg. cites/article	292	0	0	0.5	0.6	1.0	7	–
Avg. JIF	286	0.1	1.0	1.6	1.6	2.0	4.3	6
Avg. InternlFund/Faculty	151	0	769.8	2519.9	3438.5	4933.2	17501.3	141
Avg. ExternlFund/Faculty	151	0	10,779	23,912	38,926	47,141	318,941	141
Avg. TotalFund/Faculty	151	0	12,498	26,640	42,364	53,197	336,442	141
# Undergraduates	151	480	5110	8010	9556	13,104	27,005	141
# Graduates	151	0	367	770	1665	2397	11,645	141
# Faculty	164	30	393.5	707.5	1051.9	1351.0	4778.0	128
# Use in 2008	174	0	8942	48,216	200,428	178,554	3,330,858	118
# Use in 2009	178	0	9022	42,585	223,255	158,746	3,674,039	114
# Use in 2010	182	6	5356	35,246	207,557	150,640	3,463,548	110
1st author basis								
# Publications	210	1	2	9	145	83	3319	–
Times cited	210	0.0	0.00	4	127.69	58.75	3591	–
Avg. cites/article	210	0.0	0	0.5	0.5	0.8	5.0	–
Avg. JIF	207	0.1	0.9	1.4	1.4	1.8	4.0	3
Avg. InternlFund/Faculty	140	0	976.8	2743.1	3670.9	5182.7	17,501.3	70
Avg. ExternlFund/Faculty	140	0	13,449	26,304	41,382	51,217	318,941	70
Avg. TotalFund/Faculty	140	0	15,203	28,565	45,053	57,758	336,442	70
# Undergraduates	140	480	5684	8272	10,073	13,372	27,005	70
# Graduates	140	0	409.8	882.0	1772.5	2494.2	11,645	70
# Faculty	150	30	496.5	790.5	1123.4	1427.8	4778.0	60
# Use in 2008	151	0	13,466	55,567	229,570	226,906	3,330,858	59
# Use in 2009	153	0	14,995	57,351	258,492	224,985	3,674,039	57
# Use in 2010	156	6	10,387	55,324	240,913	189,648	3,463,548	54
Corresponding author basis								
# Publications	202	1.0	2.0	13.0	145.2	86.3	3178	–
Times cited	202	0.0	0.0	5.0	130.8	63.5	3539	–
Avg. InternlFund/Faculty	138	0	1156	3000	3840	5509	17,501	64
Avg. ExternlFund/Faculty	138	0	15,434	29,093	42,081	52,633	318,941	64
Avg. TotalFund/Faculty	138	0	17,631	32,166	45,921	58,962	336,442	64
# Undergraduates	138	480	6119	8748	10,434	13,824	27,005	64
# Graduates	138	0.0	452.8	1011.5	1852.4	2597.0	11,645	64
# Faculty	143	30	573	872	1198	1530	4778	59
# Use in 2008	138	0	27,027	88,192	264,961	293,350	3,330,858	64
# Use in 2009	141	0	25,406	80,704	295,960	320,525	3,674,039	61
# Use in 2010	143	8	22,912	73,904	278,153	273,844	3,463,548	59

*N*, number of samples; Min, minimum; 1st Q., first quartile; Med, median; 3rd Q., third quartile; Max, maximum; NAs, number of missing values; # Publications, number of publications in SCI(E) journals; Avg. cites/article, average cites per article; Avg. JIF, average journal impact factor; Avg. InternlFund/Faculty, average internal fund per full-time faculty/1000 KW; Avg. ExternlFund/Faculty, average external fund per full-time faculty/1000 KW; Avg. TotalFund/Faculty; Avg. InternlFund/Faculty + Avg. ExternlFund/Faculty; # Use in 2008, 2009 and 2010, number of e-journal use in 2008, 2009 and 2010, respectively

The number of publications in SCI(E) journals, times cited, and the e-journal use for 47 content providers is highly skewed to the right. Only Average JIF is close to a normal distribution with skewness and kurtosis of 0.45 and 3.41, respectively, in the co-author basis data. Times cited presents the most skewed distribution and the sharpest curve, followed by the number of articles published in SCI(E) journals. Table 1 reveals that the number of publications in SCI(E) journals, times cited and numbers for e-journal use exhibit a Pareto distribution, as is empirically observed for many natural phenomena. The highest number of publications in SCI(E) journals and the articles most cited come from only a few institutions for all author types. In addition, the majority of articles distributed through the 47 major publishers have also been used by a small number of institutions.

Due to the different sources used in the dataset, a considerable number of values are missing, as shown in Table 1. To address these discrepancies, techniques for handling missing data were applied to the analysis (described in the following section). In regards to the number of missing values, there were a lower number of missing values in institutional statistics constructed by KISTI than in institutional data constructed by KUAI and KEDI.

Table 2 details the dataset used for addressing the second question: the long term correlation analysis between research output and the use of e-journals by institutions.^5^ Only the data derived from the research output indicators based on co-author assessments were used for the long term analysis.

**Table 2:** Sample description for 10 years (2001–2010)

Variable	*N*	Min	1st Q.	Med	Mean	3rd Q.	Max	NAs
Co-author basis								
# Publications	355	1	8	32	954.9	363	38,611	–
Times cited	355	0	20	137	7613	2142	409,353	–
Avg. cites/article	355	0	2.1	4.0	4.3	6.1	13.0	–
Avg. JIF	345	0.1	1.0	1.3	1.3	1.7	4.1	10
# Use	260	0	0	24,418	494,553	243,909	12,710,776	95

The distribution of the five long term variables differs from that of the short term. The number of publications in SCI(E) journals, times cited for 355 institutions and the e-journal usage statistics for 260 institutions over 10 years exhibited a right-skewed distribution. The central peaks of these three variables also became sharper. Although more institutions have been involved in academic research output over time, only a few outstanding institutions have been responsible for the majority of research output over a long term perspective. The mean values for the number of publications, the times cited and e-journal usage statistics are higher than for the third quartiles, whereas the median values fall around the first quartiles. A few very large values for the three variables greatly influence the mean values, as seen in the short term data distribution. Two average variables, the average times cited per article and average JIF are quite close to a normal distribution in the long term dataset. The average times cited per article for 10 years exhibits a normal distribution, whereas the average times cited per article in 2010 exhibits a moderately skewed distribution. Median, mean, the first and the third quartiles, and the maximum values for average JIF over 10 years are lower than those for average JIF in 2010. The finding shows that the level of journals targeted for publication by Korean researchers has increased compared to the past. The two average variables are heavily affected by the institutions publishing a small number of articles. For example, the top-five institutions, with the exception of Pohang University of Science and Technology (POSTEC), in terms of the average times cited per article over 10 years published <50 articles and were cited <600 times, although the average times cited per article exceeded 11.6.

To address our third research question, further field-specific analysis was conducted, with the institutional statistics for articles co-authored by Korean researchers in 2010 used as shown in Table 3.

**Table 3:** Sample description in 2010 by field

WoS standard field	Variables	*N*	Min	1st Q.	Med	Mean	3rd Q.	Max	NAs
Agricultural Sciences	# Publications	148	1	2	5	17.14	18	184	–
	Times cited	148	0	0	3	12.45	15	103	–
	Avg. JIF	148	0	0.9	1.5	1.6	2.1	5.1	–
	# Use in 2010	102	1	926.8	2589.5	8298.1	8992.5	64,377	46
Arts and Humanities	# Publications	17	1	1	1	2.52	3	7	–
	Times cited	17	0	0	0	0.17	0	1	–
	Avg. JIF	17	0	0	0	0.35	1	1.16	–
	# Use in 2010	17	12	97	241	1570	914	16,449	0
Biology and Biochemistry	# Publications	150	1	2	**8**	41.33	41.25	683	–
	Times cited	150	0	1.25	**8.5**	55.67	48.50	1003	–
	Avg. JIF	150	0	2.03	2.56	2.59	3.25	5.36	–
	# Use in 2010	109	3	1522	4319	24,496	23,344	262,336	41
Chemistry	# Publications	172	1	2	**13**	**69**	53.5	978	–
	Times cited	172	0	1	**10**	**102.81**	58.25	1667	–
	Avg. JIF	172	0	1.74	2.07	2.2	2.6	5.48	–
	*# Use in 2010*	*119*	*1*	*2144*	***9788***	***56,569***	*41,228*	*580,737*	*53*
Clinical Medicine	# Publications	185	1	1	6	**84.95**	38	1947	–
	Times cited	185	0	1	4	**90.15**	42	2313	–
	Avg. JIF	185	0	1.68	2.33	2.26	2.76	5.92	–
	*# Use in 2010*	*116*	*6*	*2121*	***6327***	***49,880***	*45,649*	*576,729*	*69*
Computer Science	# Publications	140	1	1	4.5	21.04	18.25	302	–
	Times cited	140	0	0	1	6.53	5	97	–
	Avg. JIF	140	0	0.87	1.07	1.12	1.3	5.04	–
	# Use in 2010	104	26	556.2	1887	9806.7	9801	113,148	36
Economics and Business	# Publications	79	1	1	3	7.72	6.5	85	–
	Times cited	79	0	0	1	2.91	3	35	–
	Avg. JIF	79	0	0.58	1.07	1.13	1.43	5.04	–
	# Use in 2010	74	2	855	2491	6999.4	6309.2	76,418	5
Engineering	# Publications	214	1	2	**8.5**	**78.35**	50.5	1347	–
	Times cited	214	0	0	4	**69.89**	34.75	1478	–
	Avg. JIF	214	0	1.02	1.55	1.54	1.86	5.56	–
	*# Use in 2010*	*127*	*2*	*1602*	***10,046***	***60,056***	*49,402*	*738,612*	*87*
Environment/Ecology	# Publications	117	1	1	3	12.1	12	171	–
	Times cited	117	0	0	2	10.77	11	165	–
	Avg. JIF	117	0	1.4	2	2.09	2.52	4.83	–
	# Use in 2010	97	3	997	2395	9209	11,887	99,095	20
Geosciences	# Publications	110	1	1	3.5	14.5	11.75	239	–
	Times cited	110	0	1	3	17.58	11.75	273	–
	Avg. JIF	110	0	2.05	2.78	**2.71**	3.53	4.59	–
	# Use in 2010	89	2	1041	3448	11,841	11,967	117,864	21
Immunology	# Publications	81	1	1	5	12.15	15	101	–
	Times cited	81	0	1	5	13.78	18	119	–
	Avg. JIF	81	0	2.43	2.85	**3.08**	3.48	7.71	–
	# Use in 2010	72	39	520	1968	6321	8947	36,135	9
Materials Science	# Publications	153	1	2	**9**	**48.14**	43	588	–
	Times cited	153	0	1	6	**66.51**	44	1042	–
	Avg. JIF	153	0	1.49	1.92	2.20	2.48	9.86	–
	*# Use in 2010*	*112*	*1*	*1178*	***7882***	***37,443***	*28,394*	*531,908*	*41*
Mathematics	# Publications	112	1	2	6	16.12	16.25	142	–
	Times cited	112	0	0	1	7.08	7	79	–
	Avg. JIF	112	0	0.66	0.94	0.93	1.14	2.32	–
	# Use in 2010	98	13	313	818	4489	3629	53,452	14
Microbiology	# Publications	107	1	1	4	11.84	12.5	144	–
	Times cited	107	0	1	3	12.76	14.5	187	–
	Avg. JIF	107	0	1.45	2.02	2.18	2.65	6.29	–
	# Use in 2010	88	5	283.2	882.5	3668.3	4342.2	36,483	19
Molecular Biology and Genetics	# Publications	125	1	2	**8**	32.95	40	491	–
	Times cited	125	0	2	**9**	45.17	46	803	–
	Avg. JIF	125	0	2.17	2.83	**2.93**	3.62	6.29	–
	# Use in 2010	103	2	934.5	2600	16,801.3	17,394.5	169,992	22
Multidisciplinary	# Publications	50	1	1	2	4.04	4	38	–
	Times cited	50	0	0	3	20.88	24	318	–
	Avg. JIF	50	0	0.97	5.62	**8.41**	12.07	36.1	–
	*# Use in 2010*	*45*	*10*	*1888*	***8616***	*20,423*	*22,649*	*160,739*	*5*
Neuroscience and Behavior	# Publications	70	1	2	**12**	27.33	31.75	283	–
	Times cited	70	0	1	**6.5**	25.49	32.75	316	–
	Avg. JIF	70	0	2.17	2.56	2.75	3.08	7.27	–
	# Use in 2010	64	21	1354	4890	15,069	19,373	88,197	6
Pharmacology and Toxicology	# Publications	137	1	2	5	23.61	22	301	–
	Times cited	137	0	1	6	24.69	28	355	–
	Avg. JIF	137	0	1.71	2.21	2.21	2.6	5.06	–
	# Use in 2010	103	4	917.5	2777	13,728	17,141	167,973	34
Physics	# Publications	175	1	2	7	**54.49**	42.5	787	–
	Times cited	175	0	0	3	**74.4**	39.5	1437	–
	Avg. JIF	175	0	1.04	1.49	1.6	2.03	5.2	–
	*# Use in 2010*	*117*	*1*	*555*	*5129*	***32,649***	*21,339*	*437,588*	*58*
Plant and Animal Science	# Publications	114	1	2	6	20.53	19.75	341	–
	Times cited	114	0	0.25	4	14.75	14	273	–
	Avg. JIF	114	0	1.18	1.72	1.74	2.27	4.95	–
	# Use in 2010	95	3	626	2393	8811	8239	101,271	19
Psychiatry/Psychology	# Publications	85	1	1	2	7.71	9	86	–
	Times cited	85	0	0	1	5.14	5	71	–
	Avg. JIF	85	0	1.21	1.85	1.79	2.32	4.79	–
	# Use in 2010	69	43	776	1744	6440	7071	48,085	16
Social Sciences, General	# Publications	168	1	1	3	11.59	10	196	–
	Times cited	168	0	0	0	5.02	4	111	–
	Avg. JIF	168	0	0.62	1.11	1.13	1.48	3.8	–
	# Use in 2010	110	4	1063	2598	8373	7666	114,470	58
Space Science	# Publications	38	1	2	6	17.92	24	137	–
	Times cited	38	0	2.25	**17.5**	52.37	64.5	472	–
	Avg. JIF	38	0	4.37	4.96	**4.52**	4.96	6.5	–
	# Use in 2010	37	4	166	402	1722	1697	17,622	1

The highest top-five mean and median values in terms of the number of publications in SCI(E) journals, the times cited, the average JIF and numbers for e-journal usage are marked in bold. In addition, the top-five mean and median values for the e-journal usage numbers are highlighted in italics

The high performance fields in South Korea are Chemistry, Engineering, Materials Science, Molecular Biology and Genetics (three bold marks), followed by Biology and Biochemistry, Clinical Medicine, Neuroscience and Behavior, Physics and Space Science (two bold marks). The top subjects for which articles are most used by Korean researchers are Engineering, Chemistry, Materials Science, Clinical Medicine, Multidisciplinary and Physics. The fields used most are well represented as the fields in which Korean academic institutions produced the most research outcomes. Although Table 3 underlines the age-old notion that ‘the more you read, the better you write’, this study attempts to shed further light on the strong statistical relationship between e-journal use and research performance at the institution level in terms of comparative and diachronic analysis, as well as correlation analysis by field in the following sections.

### Methodology and limitations

We encountered a number of issues, as the datasets used for the study were derived from four different data sources. Each issue was dealt with as follows.

#### Identification and mapping of institution names

Due to the different sources for institutional data, the names of the institutions need to be identified first and then the data scale requires tuning for the analysis. The institutional data from KUAI and KEDI and the COUNTER JR1 are generated at a campus level whereas the research output data from NSTC reports are not. Thus the categorized statistics by campus were been summed up to derive the institutional data. For example, usage statistics for the Seoul Campus, Wonju Campus and the Medical College of Yonsei University were combined to derive the total usage statistics for Yonsei University. The names of institutions were written in the datasets originating from KUAI, KEDI and NSTC reports in Korean, whereas the names of institutions in JR1 generated by overseas publishers were written in English. In addition, some institution names change over time or the institutions may have closed when the statistics were compiled. KISTI has constructed the database of pairs of English and Korean institution names for integrating KESLI consortia information with overseas publishers. It holds the authority data on changes to institution names as well. This data was used for merging the four different data types. In addition, we searched the internet to identify the current institution if the name was not found in KISTI’s authority data.

#### Handling of missing data

As described in Tables 1, 2 and 3, a considerable number of missing values were observed in the datasets. Howell (2007) postulated that the only way to obtain an unbiased estimate is to use a model that accounts for the missing data. Such a model could then be incorporated into a more complex model for estimating missing values. In order to identify the missing values in our dataset, we examined the cause of the missing values in the institutional usage statistics.

For the exhaustive analysis, the usage statistics of 292 institutions that published at least one article in SCI(E) journals in 2010 should ideally be used to estimate the statistical relationships each other. However, only usage statistics for 182 out of 292 institutions were available for the analysis. As described in section “Dataset”, the number of publications, times cited and the numbers for e-journal use are heavily skewed to the right. Only 113 institutions achieved publication authorship exceeding 5 % in 2010, as shown in Table 4. The usage data for 111 out of 113 (98.23 %) was available for the analysis. For the long-term analysis, usage data for all 112 institutions whose authorship in SCI(E) publications exceeded 5 % were acquired.

**Table 4:** Number and proportions of institutions whose usage statistics are available

	2010			2001–2010		
	Total	Usage	Percent	Total	Usage	Percent
# Inst publishing in SCI(E)	292	182	62.33	355	260	73.24
# Inst publ. occupancy >0 %	206	159	77.19	244	205	84.02
# Inst publ. occupancy >5 %	113	111	98.23	112	112	100

# Inst publishing in SCI(E), The number of institutions publishing article(s) in SCI(E) journals; # Inst publ. occupancy >0 %, The number of institutions whose publications in SCI(E) was more than 0 %; # Inst publ. occupancy >5 %, The number of institutions whose publications in SCI(E) was more than 5 %

In general, the institutions whose usage data was not available also did not subscribe to the journals published by the 47 listed publishers, so the usage statistics for these institutions were not generated. Such institutions not subscribing to the content of major publishers are generally of low ranking for research output, as shown in Table 4. The missing values originating from KUAI and KEDI placed in low ranks as well. The two public statistics sources on higher education institutions covered the major institutions in South Korea. This fact implies that most missing values in our dataset were not random and present in the tail of the highly right-skewed distribution. In this study, issues arising from missing data were dealt with using conventional missing data techniques such as listwise deletion and mean (a representative value) substitution. For the calculation of skewness and kurtosis to identify the distribution of the variables, the missing values were omitted. We employed both listwise deletion and mean/median substitution to perform the correlation analysis between the four research output indicators and the human resources, the research funds, and the numbers for e-journal use. The missing values with the right-skewed distribution were omitted or substituted with median values. Missing data were also observed for average JIF. They were omitted or substituted with mean values, considering that the missing data was small and the distribution of average JIF is quite close to a normal distribution.

#### Mapping journal titles for subject analysis

In order to analyse the statistical relationship between the research output indicators and the numbers for e-journal usage by field, the institutional data by field were needed. The NSTC reports originate from NSI databases, and the WoS standard field is used for the subject classification system. No subject classification is assigned to the journal in COUNTER JR1 reports. Although DDC assigned by Ulrich or the British Library to journals is provided to KESLI members through KISTI’s usage statistics service, there is no mapping table between the WoS standard field and DDC. Thus, the usage statistics of journals appearing in NSTC reports are summarized at the institutional level by field. Title, P-ISSN and publisher’s names for e-journals were used as mapping keys.

#### Long-term analysis

To perform the long term analysis, two sets of institutional data have been compiled: (1) the research performance data in 2010 and e-journal usage statistics in 2008, 2009 and 2010; and (2) the research performance data and e-journal usage statistics for 10 years from 2001 to 2010. Due to the difficulties in estimating the length of time required for each researcher to review existing articles when writing a new journal article, we used the approximation that researchers use existing articles one or 2 years prior to the year of publication of the production article. The statistics for article use in 2008, 2009 and 2010 for each institution were used to investigate whether the research output in 2010 was affected by the use of e-journals during the previous 2 years. Cumulative usage statistics for each institution for 10 years were used to analyse the relationship between the research output and e-journal use from a long-term perspective.

### Limitations

COUNTER JR1 only reflects access per title in the current calendar year and provides no information about the accessed publication years as summarized in Gumpenberger et al. (2012). COUNTER JR5, which includes the usage statistics according to the publication year, was not available for the study since JR5 are only currently provided by a small number of publishers.

## Results and discussion

Pearson’s correlation coefficients (*r*) have been used to calculate the degree of the correlation between variables in the following sections.

### Comparison of e-journal usage with human resources and research funds

To analyse the statistical relationship between e-journal use and research performance by academic institutions, other institutional factors should be compared. The extent of internal/external/total research funds and the number of undergraduates/graduates/full-time faculty member at each institution were compared. As explained in section “Data, methodology and limitations”, a considerable number of missing values were observed in the institutional data. Two NA handling methods were applied in order to understand the different influences. Only the number of publications in SCI(E) journals and times cited determined from the corresponding author basis data were used to compare the strength of correlation with the seven variables.

Tables 5, 6 and 7 show that the correlations of three factors including the numbers for e-journal use, the amount of internal/external/total research funds and the number of undergraduates/graduates/full-time faculty members with the research output indicators based on co-author, first author and corresponding author, respectively, in 2010.

**Table 5:** Correlation of three factors with research performance—co-author basis

NA handling method	Factors	# Publications	Times cited	Avg. cites/article	Avg. JIF
Listwise Deletion	*# e-JournalUse*	***0.976***	***0.967***	*0.413*	*0.500*
	Avg. InternlFund/Faculty	0.389	0.374	0.470	0.501
	Avg. ExternlFund/Faculty	0.572	0.585	0.476	0.572
	Avg. TotalFund/Faculty	0.574	0.586	**0.488**	**0.582**
	# Undergraduates	0.581	0.500	0.294	0.308
	# Graduates	0.878	0.837	0.427	0.463
	# Faculty	0.833	0.780	0.417	0.469
Mean/median substitution	*# e-JournalUse*	***0.976***	***0.971***	*0.226*	***0.393***
	Avg. InternlFund/Faculty	0.428	0.406	0.239	0.338
	Avg. ExternlFund/Faculty	0.596	0.600	**0.243**	0.389
	Avg. TotalFund/Faculty	0.546	0.558	0.219	0.327
	# Undergraduate	0.588	0.512	0.180	0.231
	# Graduate	0.863	0.822	0.227	0.347
	# Faculty	0.772	0.727	0.211	0.276

Significant at *p* < 0.05. The highest values are marked in bold. The *r* value between the four research indicators and the numbers for e-journal use are highlighted in italics

**Table 6:** Correlation of three factors with research performance—first author basis

NA handling method	Factors	# Publications	Times cited	Avg. cites/article	Avg. JIF
Listwise Deletion	*# e-JournalUse*	***0.976***	***0.966***	*0.307*	*0.467*
	Avg. InternlFund/Faculty	0.384	0.376	0.340	0.418
	Avg. ExternlFund/Faculty	0.567	0.601	0.354	0.491
	Avg. TotalFund/Faculty	0.569	0.601	**0.363**	**0.499**
	# Undergraduates	0.571	0.486	0.200	0.205
	# Graduates	0.875	0.819	0.269	0.404
	# Faculty	0.824	0.766	0.288	0.364
Mean/median substitution	*# e-JournalUse*	***0.978***	***0.968***	***0.254***	***0.439***
	Avg. InternlFund/Faculty	0.370	0.355	0.228	0.306
	Avg. ExternlFund/Faculty	0.542	0.562	0.229	0.357
	Avg. TotalFund/Faculty	0.543	0.562	0.235	0.363
	# Undergraduates	0.542	0.457	0.151	0.170
	# Graduates	0.822	0.758	0.179	0.295
	# Faculty	0.770	0.702	0.205	0.272

**Table 7:** Correlation of three factors with research performance—corresponding author basis

NA handling method	Factors	# Publications	Times cited
Listwise deletion	*# e-JournalUse*	***0.956***	***0.946***
	Avg. InternlFund/Faculty	0.358	0.353
	Avg. ExternlFund/Faculty	0.591	0.626
	Avg. TotalFund/Faculty	0.590	0.623
	# Undergraduates	0.543	0.461
	# Graduates	0.866	0.812
	# Faculty	0.806	0.750
Mean/median substitution	*# e-JournalUse*	***0.960***	***0.951***
	Avg. InternlFund/Faculty	0.347	0.335
	Avg. ExternlFund/Faculty	0.561	0.582
	Avg. TotalFund/Faculty	0.561	0.579
	# Undergraduates	0.516	0.433
	# Graduates	0.813	0.750
	# Faculty	0.748	0.682

External funds have a stronger correlation than Internal funds because academic institutions with better research performance tend to receive more funds from the government or industry in general. Unexpectedly, the number of graduate students had a strong statistical relationship with the two quantitative research performance indicators and ‘average cites per articles’ compared to the number of faculty members in academic institutions, regardless of the author-type and the NA handling method. The number of faculty had a stronger correlation with ‘average JIF’ only in co-author basis data when missing data were omitted, when compared to the number of graduates. However, the difference is quite slight at 0.003, thus it does not seem to be significant. e-Journal use and the number of graduates and full-time faculty members shows a strong relationship with the number of publications in SCI(E) journals and times cited, whereas the average extent of external/total funding per faculty member, and the number of undergraduates show moderate correlation values with the two indicators.

With two qualitative indicators including ‘average cites per article’ and ‘average JIF’, the total amount of research funds had the highest correlation in co-author and first author basis data when the missing data were omitted.

The first author basis data showed similar results to the co-author basis data, except that the correlation value of e-journal use is highest when the missing data are substituted with the mean or median values, as shown in Table 6. The four research performance indicators had the highest correlation with the number of e-journal use in first author basis data set when the missing values were substituted with mean or median values.

As explained in section “Data, methodology and limitations”, two research performance indicators were available for the correlation analysis in corresponding author basis data as presented in Table 7.

The association of the seven independant variables with two indicators did not differ in terms of the corresponding author data from the two previous author types.

**Fig. 1:**
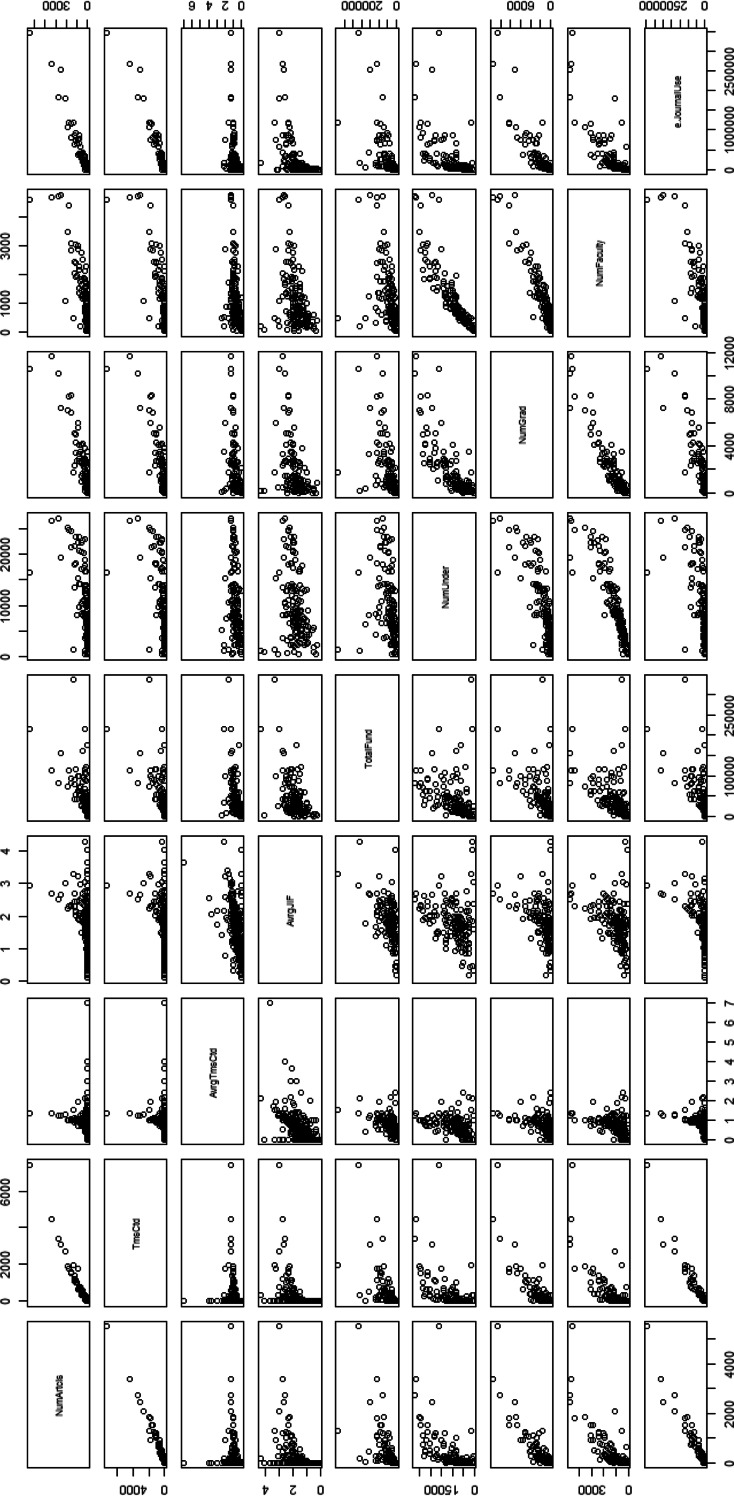
Pairs plot for 9 variables with listwise deletion-co-author basis

**Fig. 2:**
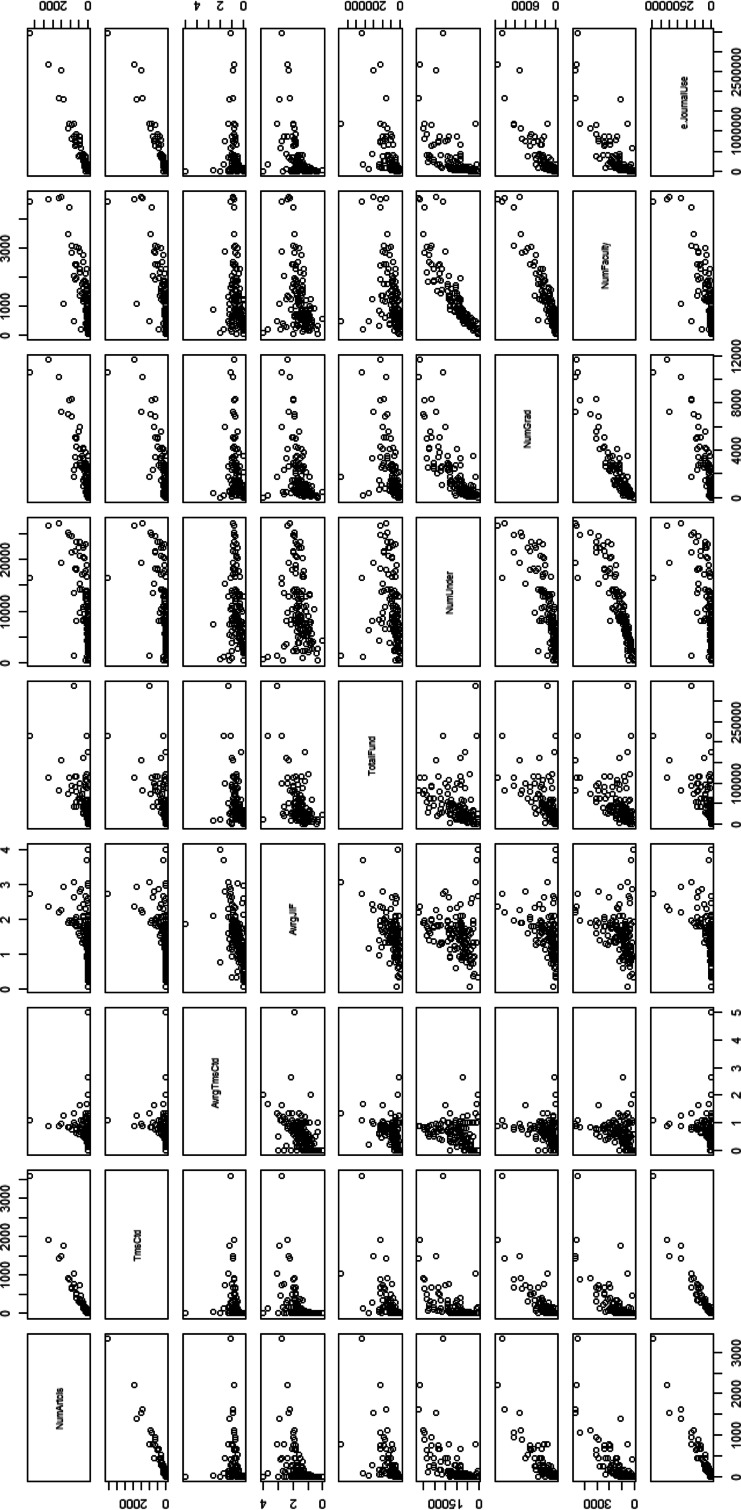
Pairs plot for 9 variables with mean/median substitution-co-author basis

E-journal use shows the highest correlation with two quantitative performance indicators ‘# Publication’ and ‘Times cited’, regardless of NA handling method and author type, as shown in Tables 6, 7 and 8. In other words, the number of articles published in SCI indexed journals and the times cited are more strongly correlated with e-journal use at the academic institution level than with the economic factors and number of researchers. With regard to the average times cited per article, e-journal usage has the highest correlation only with the first author basis data when NAs are substituted with mean or median values. With the average JIF, e-journal usage shows the highest values in the co-author and the first author basis data only when NAs are substituted with mean or median values. However, the differences between the maximum values and that of the correlation with e-journal use using two average indicators were not significant. Statistically, the accountability of e-journal use for the average times cited per article and the average JIF is quite close to that of the total extent of research funds. Moreover, the accountability of e-journal use for the two qualitative indicators is better than that for the number of graduates or the number of faculty members in two author data types. Figure 1 presents the pairs plot for variables, including the four outcome indicators and the seven variables when missing data are omitted. Figure 2 presents the plot with mean/median substitution for co-author basis data.

**Table 8:** Pearson’s correlation coefficients between four research output indicators in 2010 and e-journal use in each time-window, according two NA handling methods and three author types

Author type	NA handling method	Factor (# use)	# Publications	Times cited	Avg. cites/article	Avg. JIF
Co-author	Listwise deletion	2008	0.927	0.929	0.394	0.476
		2009	0.957	0.953	0.406	0.493
		2010	**0.976**	0.967	**0.413**	**0.500**
		2008–2009	0.946	0.944	0.400	0.487
		2009–2010	0.971	0.966	0.412	0.499
		2008–2010	0.962	0.959	0.406	0.494
	Median substitution	2008	0.929	0.932	0.217	0.373
		2009	0.959	0.955	0.223	0.387
		2010	**0.976**	**0.971**	0.226	0.393
		2008–2009	0.948	0.947	0.221	0.382
		2009–2010	0.972	0.968	0.225	0.392
		2008–2010	0.964	0.961	0.224	0.388
First author	Listwise deletion	2008	0.925	0.922	0.288	0.437
		2009	0.957	0.946	0.297	0.454
		2010	0.976	0.966	**0.307**	**0.467**
		2008–2009	0.945	0.938	0.294	0.447
		2009–2010	0.972	0.961	0.304	0.463
		2008–2010	0.962	0.954	0.301	0.457
	Median substitution	2008	0.932	0.926	0.238	0.413
		2009	0.962	0.952	0.249	0.431
		2010	**0.978**	**0.968**	0.254	0.439
		2008–2009	0.951	0.942	0.245	0.424
		2009–2010	0.975	0.965	0.252	0.437
		2008–2010	0.966	0.957	0.249	0.432
Corresponding author	Listwise deletion	2008	0.908	0.906		
		2009	0.938	0.928		
		2010	**0.956**	**0.946**		
		2008–2009	0.927	0.921		
		2009–2010	0.953	0.943		
		2008–2010	0.944	0.936		
	Median substitution	2008	0.916	0.910		
		2009	0.945	0.936		
		2010	**0.960**	**0.951**		
		2008–2009	0.935	0.927		
		2009–2010	0.958	0.948		
		2008–2010	0.950	0.941		

Significant at *p* < 0.05. The highest values are marked in bold

As seen in Fig. 1, the statistics for the number of e-journals exhibit some degree of correlation with the two average research output indicators when missing data are omitted. The NA handling method chosen influenced correlation with the two average variables. As described in section “Methodology and limitations”, many of the statistics for e-journal use at institutions that published a lower than average number of publications are missing. In section “Dataset” shows that the average times cited per article and average JIF have been heavily influenced by institutions that publish a relatively small number of articles. With listwise deletion, many of those institutions that published fewer publications become omitted. This results in stronger correlations between the statistics for article use and the two average indicators for institutions that remain in the analysis.

### Long-term relationship between article use and research performance

We investigated the statistical relationship between the research output indicators and the article use indicator at the institutional level over an extended period of time. As explained in section “Data, methodology and limitations”, missing values were observed in the average JIF and the institutional e-journal usage statistics. Six do not have published data for average JIF in 2010 and ten institutions do not have 10-year data. There are 118, 114, 110 and 95 institutions that did not release statistics for e-journal use in 2008, 2009, 2010 and during the 10-year period, respectively. The missing values for average JIF have been replaced with mean values, whereas the numbers for e-journal use are replaced with their median values if the mean/median substitution technique is applied.

#### Correlation between e-journal use in 2008, 2009, 2010 and four research output indicators in 2010

The association between the usage of scholarly information over previous years and current research performance indicators has been examined. Due to the difficulties in estimating the length of time required for each researcher to review existing articles when writing a new journal article, we used the approximation that researchers use existing articles one or 2 years prior to the year of publication of the production article. The statistics for e-journal use in 2008, 2009 and 2010 for each institution were used to investigate whether the research output in 2010 was affected by the use of e-journals during the previous years, including 2010. Table 8 presents the correlation of e-journal use in each time window (1 year, 2 years, 3 years) with four research output indicators calculated based on three types of author according to two NA handling methods.

If medians are replaced for e-journal usage numbers, the correlation increases with the two counting variables slightly for each year, and the time-window (2, 3 years) when compared to listwise deletion. The correlation between e-journal use and the two average variables of research output are slightly stronger when listwise deletion is applied.

In contrast to what was assumed, the statistics for article use in the prior one and 2 years did not appear more effect on the research output in 2010. The numbers for e-journal use in 2010 exhibited the strongest correlation with the four research output indicators in 2010, regardless of the NA handling method used.

#### Correlation between total use and four research output indicators for 10 years

Association between e-journal use and the four research performance indicators of each institution for an extended time period has also been examined as well. Only co-author basis data on research output for 10 years are used. Pearson’s correlation coefficients (*r*) between four research output indicators and the article use indicator from 2001 to 2010 are presented in Table 9.

**Table 9:** Pearson’s correlation coefficients between four research output indicators for 10 years and article use in relation to co-author basis as the NA handling method

NA handling method	# Publications	Times cited	Avg. cites/article	Avg. JIF
Listwise deletion	0.937	0.929	0.447	0.447
Median substitution	0.939	0.931	0.398	0.392

Significant at *p* < 0.05

The analysis of long-term data shows that the correlation between the numbers for e-journal use and the number of publications in SCI(E) journals and times cited is weaker than that for short-term data, whereas the *r* values between the numbers for e-journal use and the average cites per article are higher in the long-term data regardless of the NA handling method. The difference in correlation values according to NA handling method is small for the long-term data whereas the handling method affected correlations for article use with the two average variables in the shorter-term data.

In conclusion, the numbers for e-journal usage retains a strong positive correlation (*r* > 0.9, *p* < 0.05) with the number of articles published in SCI(E) journals and times cited from a long term perspective, in addition to the fact that the *r* values between the numbers for e-journal use and the average cites per articles are higher in the long-term data than for the short-term data regardless of the NA handling method used.

#### Star plots of five indicators for institutions in 2010 and for the years 2001–2010 (10 years)

**Fig. 3:**
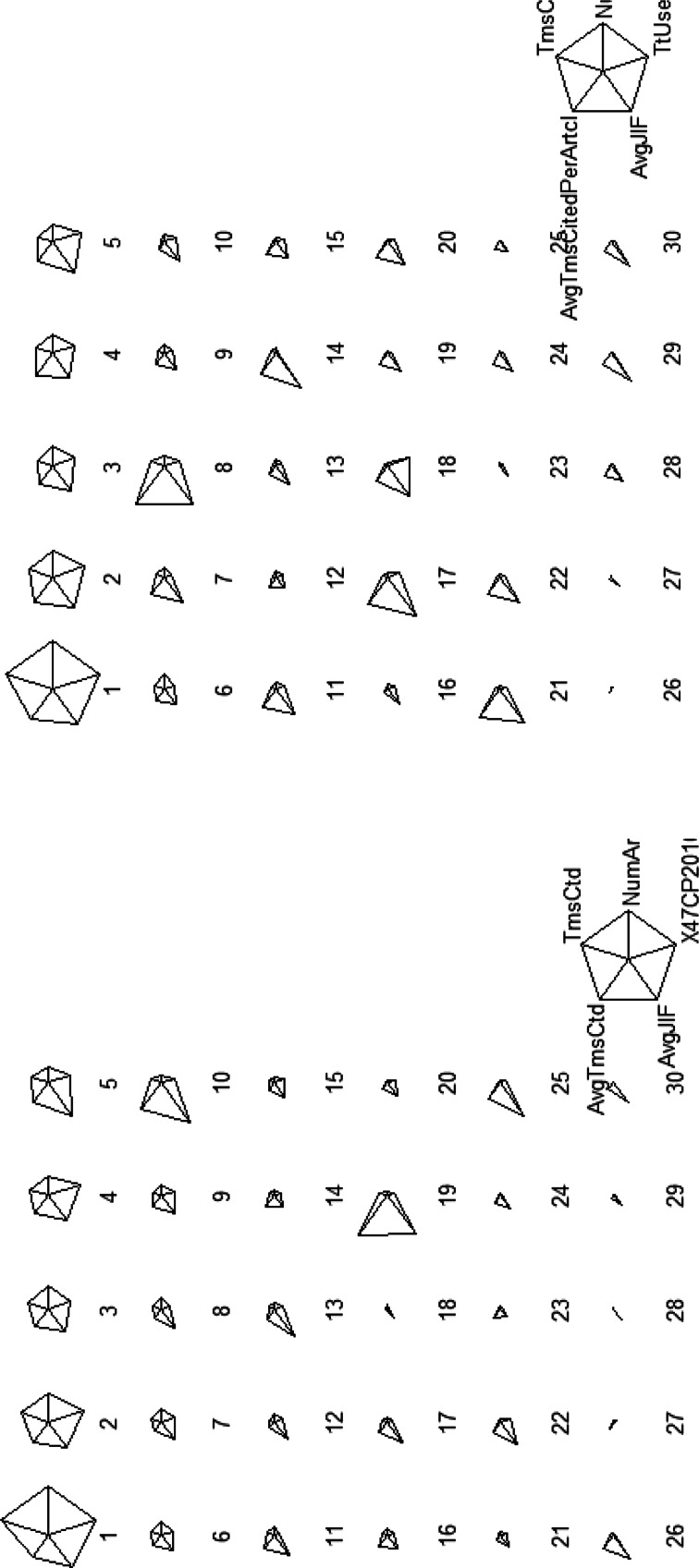
Star plots of five indicators for 2010 (*left*) and for 2001–2010 (*right*)

Star plots for four research output indicators derived from the co-author basis data and e-journal usage at the institutional level are illustrated in Fig. 3, detailing scholarly activities for the 1-year and 10-year periods. Each star represents a single institution in South Korea. The stars are arranged in order of the highest number of publications in SCI(E) journals.

As seen in Fig. 3, data from the top five institutes produce star plots that take the shape of full pentagons. From the sixth to the tenth institute, the size of the stars decreases, with one axis receding disproportionately. The following stars lose their pentagonal shape and some become triangular or single lines. It can be seen that the top five institutions are performing evenly in terms of article publications in SCI(E) journals with high IFs and being highly cited, and using a considerable number of existing publications. Specific statistics for the five variables over 10 years for the top-ten institutions are shown in Table 10.

**Table 10:** Publications, citations and total use statistics for the top-ten institutions publishing the most articles in S. Korea. (2001–2010)

Institution	Publications		Citations			e-Journal use^a^
	Number	Percent	Times cited	Avg. cites/article	Avg. JIF	
Seoul Nat. Univ.	38,611	14.28	409,353	10.60	2.535	8.56
Yonsei Univ.	21,546	7.97	210,915	9.79	2.387	5.93
Korea Univ.	17,214	6.37	145,562	8.46	2.251	4.02
KAIST	16,770	6.20	155,057	9.25	2.192	4.14
Sungkyungwan Univ.	15,795	5.84	135,633	8.59	2.401	5.46
Hanyang Univ.	13,472	4.98	96,053	7.13	1.902	2.02
Kyungpook Univ.	10,336	3.82	86,083	8.33	2.227	1.38
POSTECH	10,159	3.76	125,915	12.39	2.662	2.89
Pusan Nat. Univ.	9960	3.68	71,025	7.13	1.921	1.67
Kyunghee Univ.	8180	3.02	52,557	6.43	2.038	2.24

^a^Scaled figures for each institution’s usage have been presented, as actual usage data is confidential. Scaling was conducted by dividing centered usage statistics by their standard deviations, or the root mean square

The listed institutions in Table 10 are also regarded as top South Korean universities in terms of the number of qualified faculty and the size of research funds.

### Relationship between article use and research output by subject

We analyzed the statistical relationship between the research output indicators including the number of publications in SCI(E) journals, times cited and average JIF, and e-journal use by field. Table 11 presents the correlation values for e-journal usage with the three research output indicators in 23 WoS standard fields.

**Table 11:** Correlation of e-journal use with three research performance indicators by field

WoS standard field	# Publications	Times cited	Avg. JIF
Agricultural Sciences	0.92	0.89	0.04
Arts and Humanities	−0.06	−0.11	0.21
Biology and Biochemistry	0.92	0.90	0.34
Chemistry	0.88	0.85	0.48
Clinical Medicine	0.92	**0.91**	0.09
Computer Science	**0.96**	0.90	−0.06
Economics and Business	0.89	0.81	0.01
Engineering	0.86	0.87	0.32
Environment/Ecology	0.90	0.85	0.13
Geosciences	0.78	0.81	0.11
Immunology	0.91	**0.91**	0.09
Materials Science	0.86	0.84	0.23
Mathematics	0.86	0.81	0.25
Microbiology	0.91	0.89	0.17
Molecular Biology and Genetics	0.91	0.90	0.29
Multidisciplinary	0.89	0.78	0.31
Neuroscience and Behavior	0.85	0.81	0.06
Pharmacology and Toxicology	0.76	0.74	0.16
Physics	0.89	0.88	**0.52**
Plant and Animal Science	0.91	0.90	0.07
Psychiatry/Psychology	0.94	0.90	0.15
Social Sciences, general	**0.96**	**0.91**	0.08
Space Science	0.85	0.78	0.14

Significant at *p* < 0.05. The highest values are marked in bold

**Fig. 4:**
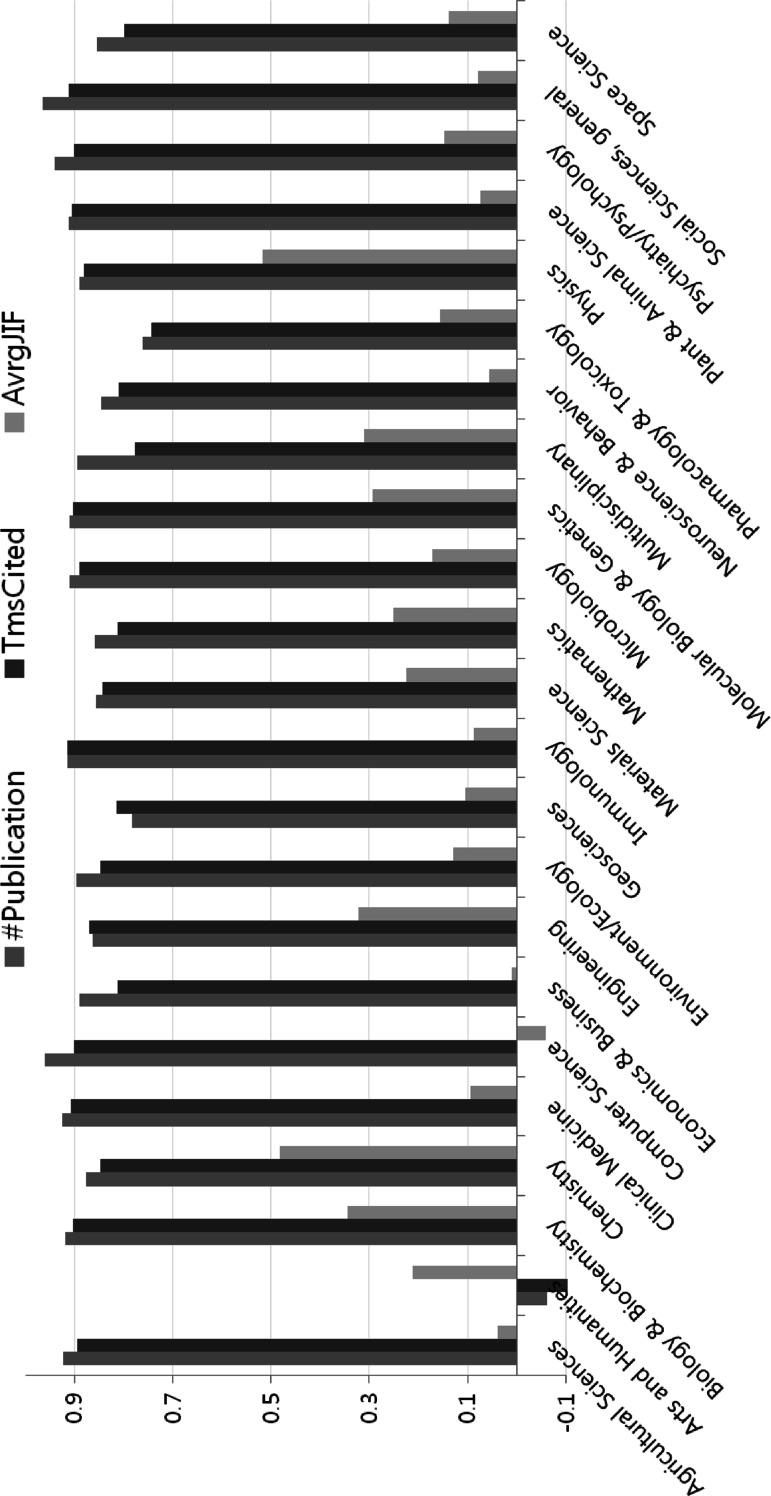
Correlation between e-journal usage and three research performance indicators by field

The correlation coefficient values for e-journal use with research performance indicators differ by field. The *r* value for e-journal use with the number of publications varies dramatically from −0.06 to 0.96. The highest scoring fields are Computer Science and Social Sciences, general in terms of the correlation of e-journal use with the number of publications in SCI(E) journals. The lowest field is Arts and Humanities. Clinical Medicine, Immunology and Social Sciences, general have the highest *r* value between e-journal use and times cited. The highest correlation value, 0.52, between e-journal use and average JIF is presented in Physics. The weakest relationship between the numbers for e-journal use and average JIF was for Computer Science which has the strongest association between e-journal usage and publications in SCI(E) journals. The degree of association between e-journal use and research output at each institution by field did not correlate with the strength of research performance by field. However, the numbers for e-journal use had a strong positive correlation with the number of publications in SCI(E) journals and the times cited in every WoS standard field, except the Arts and Humanities, as illustrated in Fig. 4.

In this study, we observed that measures of research article use had a strong positive relationship with two research output indicators and approximately medium correlations with the two average indicators in our institutional dataset, regardless of the time-period or the subject field. In the comparative analysis, the numbers for e-journal use had the strongest association with the number of publications in SCI(E) journals and the times cited than measures for human resources or research funds. The difference in *r* for e-journal use with two average values on research output quality was not significant from that of the extent of external fund per faculty (which had the highest value).

Miller (1992) concluded that the combination of organizational and bibliometric indicators offered a valid option to assess the quality of research produced by research organizations. We suggest that the numbers for e-journal use by institution may be included in organizational data or as indicators for assessing the institutions. We expect that the number of articles used may function as a more direct and reliable indicator for estimating research performance at each institution.

## Conclusions and further work

In this study, we explored the statistical relationship between research output and e-journal usage at institutions in South Korea by performing the comparative and diachronic analyses, and the analysis by field. Three sets of data according to author type were compiled for the comparative analysis and the diachronic analysis. Due to the different data sources utilized for the analyses, a considerable number of missing values appeared in our datasets and the mapping issues had to be solved prior to the analysis. Two techniques for handling missing data were applied and the effect of each technique was discussed. In order to analyse the institutional data by field, journals were mapped first, and the statistics were then summarized according to subject field.

We found that the distribution for number of times cited, the number of articles published in SCI(E) journals and the number of articles used by institutions was highly skewed to the right, whereas average JIF values exhibited an almost normal distribution. The distribution of average times cited per article was slightly skewed to the right in the 1 year data, however, the distribution of the same variable in the 10-year dataset was reasonably close to a normal distribution. In addition, we investigated the statistical relationship between research output indicators and article use with short- and long-term datasets. Although the considerable amount of missing data was problematic, we have identified the missing data and have applied two NA handling methods to calculate the correlation between the four research output indicators and article use. As a result, we observed that e-journal usage showed a stronger correlation with the number of publications and the times cited regardless of NA handling method or author type compared to the number of undergraduates, graduates, faculty members and the extent of research funding. The differences between the maximum correlation values for average external research funding per full-time faculty with two average indicators and e-journal usage was not significant. Statistically, the accountability of e-journal usage for the average times cited per article and the average JIF was quite close to that of the amount of external research funding. It was found that the statistics for article use exhibited a strong positive correlation with the number of articles published in SCI(E) journals and the times cited regardless of the author type, time period, subject category and NA handling method. The average times cited per article and average JIF are heavily influenced by the institutions that publish lower numbers of articles. This has resulted in differences in correlations between the total articles used and the two average variables, depending on the time period and NA handling method employed. With median substitution, correlations for usage numbers, average times cited per article and average JIF are relatively weak, whereas with listwise deletion, correlations between them are improved when analyzing short term data. However, differences due to the NA handling method in correlation with article use and the two average variables in the long-term data was not significant. We observed that the top-five institutions in South Korea, with respect to the number of publications in SCI(E) journals, generally engage in a balance across the types of academic specialties, while producing outstanding research output and using existing publications. Finally, we confirmed that the association of e-journal use with the two quantitative research indicators is strong in the analysis by field, with the exception of the Arts and Humanities. These results may be utilized to predict trends in research and development at the institutional level and the country level.

From the viewpoint of science policy studies, trends in research are of significant importance (Vinkler 2010). The identification and prediction of emerging or declining research fields by tracking the use of articles by subject may contribute as informative tools for science policy-makers. We intend to conduct a further study at the journal level with identical data sources to address this question. Furthermore, it would be of interest to explore the relationship between research output and article use at different levels, such as that of the individual, research group, nation or region, and these investigations could take into account various indicators and data input sources. If a strong relationship between research output and article use is found in general, then the usage data could conceivably contribute to a better understanding of scholarly communication, activities and impact.

## Footnotes

1 The average JIF for each institution is based on the Journal Citation Report published by the WoS according to publication year, and is compiled by the NSTC.

2
http://aims.kcue.or.kr/.

3
http://kess.kedi.re.kr/index.

4
http://www.projectcounter.org/r3/Release3D9.pdf.

5 Past data for the number of undergraduates, graduates, faculty members and the average internal/external research fund per faculty member were not available for the long term study. The institutional statistics before 2010 from KUAI and KESS are currently not available to the public.
